# Ten emerging SARS-CoV-2 spike variants exhibit variable infectivity, animal tropism, and antibody neutralization

**DOI:** 10.1038/s42003-021-02728-4

**Published:** 2021-10-13

**Authors:** Li Zhang, Zhimin Cui, Qianqian Li, Bo Wang, Yuanling Yu, Jiajing Wu, Jianhui Nie, Ruxia Ding, Haixin Wang, Yue Zhang, Shuo Liu, Zhihai Chen, Yaqing He, Xiaodong Su, Wenbo Xu, Weijin Huang, Youchun Wang

**Affiliations:** 1 Division of HIV/AIDS and Sex-transmitted Virus Vaccines, Institute for Biological Product Control, National Institutes for Food and Drug Control (NIFDC) and WHO Collaborating Center for Standardization and Evaluation of Biologicals, Beijing, China; 2 Jiangsu Recbio Technology Co., Ltd., Taizhou, China; 3 Beijing Advanced Innovation Center for Genomics (ICG) & Biomedical Pioneering Innovation Center (BIOPIC), Peking University; State Key Laboratory of Protein and Plant Gene Research, School of Life Sciences, Peking University, Beijing, China; 4 Institute of Infectious Diseases, Beijing Ditan Hospital, Capital Medical University, Beijing, China; 5 Shenzhen Center for Disease Control and Prevention, Shenzhen, Guangdong China; 6 National Institute for Viral Disease Control and Prevention, Chinese Center for Disease Control and Prevention, Beijing, China

**Keywords:** SARS-CoV-2, Viral infection

## Abstract

Emerging mutations in SARS-CoV-2 cause several waves of COVID-19 pandemic. Here we investigate the infectivity and antigenicity of ten emerging SARS-CoV-2 variants—B.1.1.298, B.1.1.7(Alpha), B.1.351(Beta), P.1(Gamma), P.2(Zeta), B.1.429(Epsilon), B.1.525(Eta), B.1.526-1(Iota), B.1.526-2(Iota), B.1.1.318—and seven corresponding single amino acid mutations in the receptor-binding domain using SARS-CoV-2 pseudovirus. The results indicate that the pseudovirus of most of the SARS-CoV-2 variants (except B.1.1.298) display slightly increased infectivity in human and monkey cell lines, especially B.1.351, B.1.525 and B.1.526 in Calu-3 cells. The K417N/T, N501Y, or E484K-carrying variants exhibit significantly increased abilities to infect mouse ACE2-overexpressing cells. The activities of furin, TMPRSS2, and cathepsin L are increased against most of the variants. RBD amino acid mutations comprising K417T/N, L452R, Y453F, S477N, E484K, and N501Y cause significant immune escape from 11 of 13 monoclonal antibodies. However, the resistance to neutralization by convalescent serum or vaccines elicited serum is mainly caused by the E484K mutation. The convalescent serum from B.1.1.7- and B.1.351-infected patients neutralized the variants themselves better than other SARS-CoV-2 variants. Our study provides insights regarding therapeutic antibodies and vaccines, and highlights the importance of E484K mutation.

## Abstract (web summary)

Li Zhang, Zhimin Cui, and Qianqian Li et al. compare the infectivity, host tropism, and antigenicity of 10 SARS-CoV-2 variants using a VSV-based pseudovirus system. Their results suggest that variants carrying E484K display the most significant reduction in sensitivity to neutralization, and may provide further insight into the development of relevant therapeutics for SARS-CoV-2 infection.

## Introduction

**Fig. 1:**
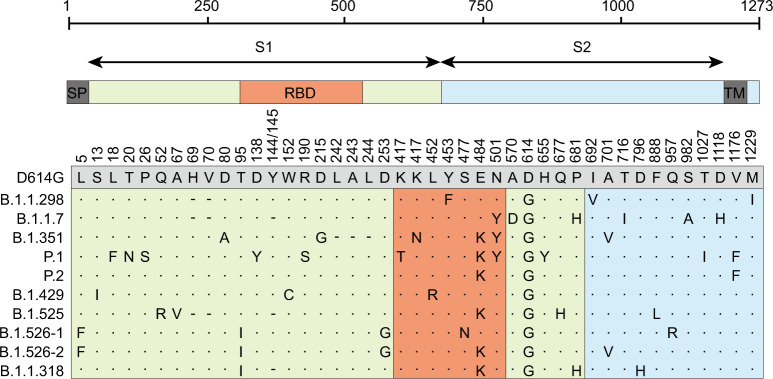
Schematic of SARS-CoV-2 variants. The most representative amino acid mutations of each variant were selected to construct pseudotyped virus for this study. Each mutation in each SARS-CoV-2 variant is indicated relative to the reference D614G sequence. A dot indicates an identical amino acid at the indicated position, while a dash indicates a deletion at that point. SP sigmal peptide, TM transmembrane domain, RBD Receptor-binding domain.

Following the discovery of SARS-CoV-2, the emergence of multiple variants has been reported^1,2^. Mutations of the virus may cause changes in its infectivity and pathogenicity, resulting in the emergence of highly infectious or lethal mutant strains, they may also change the antigenicity of the virus, leading to failures of existing antibody treatments or the vaccine^1,3,4^. Additionally, mutations may cause cross-species transmission and the virus may undergo further evolution in the new host, triggering a new wave of virus spread^4^. Therefore, the mutations of SARS-CoV-2 have received close attention from scientists worldwide. Beginning in March 2020, the D614G mutant strain became the dominant strain globally, and the current prevalence has exceeded 95%^3^. In November 2020, the mink strain B.1.1.298 (cluster 5) was reported to spread between humans and minks^4,5^. Since December, increasing numbers of SARS-CoV-2 variants have been reported worldwide, among which B.1.1.7, B.1.351, and P.1 have been listed as viruses of concern (VOCs) by the WHO^6^. As of March 2021, B.1.1.7(alpha, VOC 202012/01 or 501Y.V1), which first appeared in the United Kingdom, has spread to 125 countries; this variant exhibits increased transmissibility, risk of hospitalization, severity, and mortality^7–9^. B.1.351(beta or 501Y.V2) first appeared in South Africa and leads to immune escape of the spike protein because of mutation E484K in the RBD; this variant may influence the efficacies of vaccines and therapeutic monoclonal antibodies (mAbs) and sera^10,11^. P.1 (gamma, B.1.1.28.1, or 501Y.V3) and P.2 (Zeta), which first appeared in Brazil, led to the disappointment regarding Brazil’s herd immunity dream and almost caused the collapse of Brazil’s medical system^12,13^. Variants B.1.429 (Epsilon or B.1.427) in California and B.1.526 (Iota) in New York comprised the largest proportion of the new COVID-19 cases in those areas, eliciting widespread concern^1,14–17^. Moreover, the Nigerian variant B.1.525 (Eta), which contains subsets of mutations previously observed in variants B.1.1.7 and B.1.351, has spread rapidly in Nigerian and the United Kingdom^18,19^. Additionally, a new variant B.1.1.318 recently appeared in the United Kingdom; this variant requires close attention because of its E484K mutation^18,20^. In this study, we investigated the 10 currently prevalent variants (B.1.1.298, B.1.1.7, B.1.351, P.1, P.2, B.1.429, B. 1.525, B.1.526-1, B.1.526-2, and B.1.1.318) using the VSV-based pseudovirus system (Fig. 1). We compared infectivity, host tropism, and neutralization characteristics with the D614G reference strain, with the aim of providing clues for the prevention and control of COVID-19, particularly with respect to designing mAbs and vaccines.

## Results

### Infectivities of 10 SARS-CoV-2 variants

**Fig. 2:**
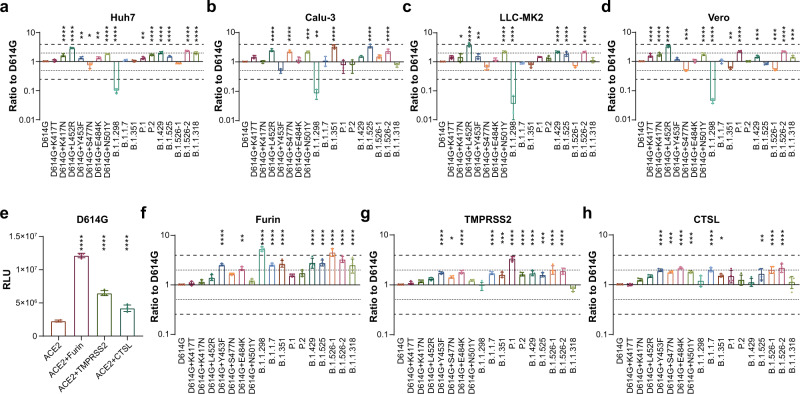
The infectivity of SARS-CoV-2 Variants in primate cell lines and Furin/TMPRSS2/CTSL expressed 293T-ACE2 cells. **a**–**d** SARS-CoV-2-susceptible cells as indicated were used to compare the infectivities among 10 SARS-CoV-2 variants. Pseudostyped virus was quantified via RT-PCR and all strains were diluted to the same copy number. Cells were harvested 24 h after pseudostyped virus infection and analyzed for luminescence activities (RLUs). Relative infectivities compared with the D614G reference strain are displayed as the RLU ratio to D614G. **e**–**h** furin, TMPRSS2, and cathepsin L (CTSL)-stably overexpressing 293T-hACE2 cells were used compared to mock 293T-hACE2 cells. All cells were infected with the same copy number of pseudotyped SARS-CoV-2 variants. Chemiluminescence signals were detected 24 h later. Data shown indicate relative changes in infectivity due to the enzyme overexpression. Relative RLUs were compared with or without the indicated enzyme first, and then compared with the D614G reference strain. **e** RLU of cells infected with D614G virus. **f**–**h** Ratios by which enzyme overexpression induced enhanced infection, compared with D614G. The results were obtained from four independent experiments and the values shown indicate means ± SEMs. Dashed/dotted lines indicate the threshold of fourfold/twofold difference. Asterisks indicate statistical significance.

The infectivities of the 10 variants and seven RBD-located single mutations were first tested in four SARS-CoV-2-susceptible cell lines, including two human cell lines (Huh-7, and Calu-3) and two non-human primate cell lines (LLC-MK2 and Vero). Notably, although most of the examined SARS-CoV-2 variants showed slightly increased infectivity, none of them had more than fourfold increased infectivity, compared with the D614G reference strain (Fig. 2a–d). The L452R single mutation and B.1.526-2 led to increased infectivity, whereas the B.1.1.298 variant exhibited significantly decreased infectivity in all the four cell lines (Fig. 2a–d). Moreover, the variants B.1.351 B.1.525 and B.1.526-2(E484K) showed significantly increased infectivity for Calu-3 cells (Fig. 2b). We further analyzed the reason for decreased infectivity of B.1.1.298. The single mutations M1229I may be the key mutation that caused the decreased infectivity of B.1.1.298 (Supplementary Fig. 1), while the expression level of B.1.1.298 spike protein was significantly decreased compared to D614G mutation, which may be responsible for the observed reduced infection (Supplementary Fig. 2).

### The activity of furin/TMPRSS2/cathepsin L the ten SARS-CoV-2 variants

Because furin, TMPRSS2, and cathepsin L play important roles in SARS-CoV-2 infection^21–23^, mutation-related structural changes in the virus may influence the functions of these enzymes. We found that the ability of the D614G reference strain to infect 293T-ACE2 cells was significantly increased when furin, TMPRSS2, or cathepsin L was overexpressed (Fig. 2e). We subsequently investigated the infectivities of the variants in respective furin-, TMPRSS2-, or cathepsin L stable overexpressing cells. The results showed that the increased infectivity to the 293T-ACE2 cells in the presence of furin, TMPRSS2, or cathepsin L was further increased among most of the tested variants, excluding B.1.1.298 and B.1.1.318 in the TMPRSS2 group, as compared to the D614G reference strain (Fig. 2f–h).

### Animal tropism of 10 SARS-CoV-2 variants

**Fig. 3:**
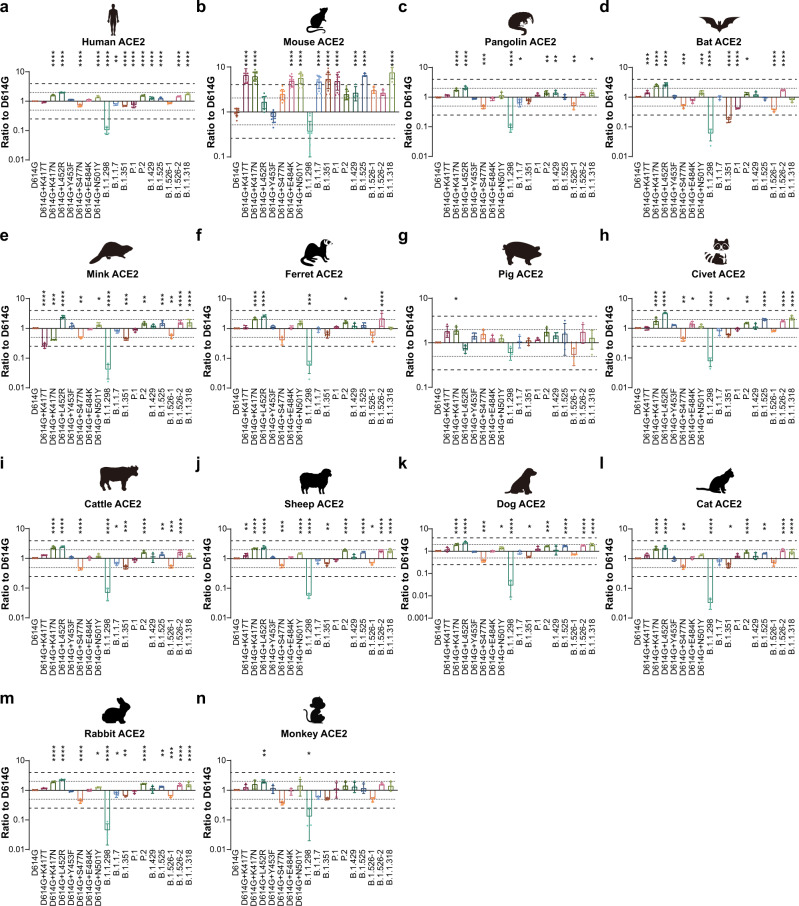
Infectivities of SARS-CoV-2 Variants in cell lines expressing ACE2 proteins from 14 different species. Equal amounts of overexpression plasmids carrying ACE2 sequences from different species were transfected into 293 T cells. Transfection efficiency was confirmed by flow cytometry analysis of ACE2 surface expression. Cells were then infected with the same copy number of SARS-CoV-2 variants after quantification. Chemiluminescence signals were collected 24 h later. Ratios between variants and the D614G reference strain were calculated. The results were obtained from four independent experiments and the values shown indicate means ± SEMs. **a** human; **b** mouse; **c** pangolin; **d** bat; **e** mink; **f** ferret; **g** pig; **h** civet; **i** cattle; **j** sheep; **k** dog; **l** cat; **m** rabbit; **n** monkey. Dashed/dotted lines indicate the threshold of fourfold/twofold difference. Asterisks indicate statistical significance.

To investigate changes in the animal tropism of the 10 SARS-CoV-2 variants, 14 ACE2s from different species were overexpressed in 293 T cells. The infectivities of most variants were increased in the mouse ACE2 overexpressed cell lines (Fig. 3b). Notably, the infectivities of the B.1.1.7, B.1.351, P.1, B.1.525, and B.1.1.318 were significantly increased (by more than fourfold), compared with the D614G reference strain (Fig. 3b). Single mutation analyses showed that K417T, K417N, E484K, and N501Y led to increased infectivity in mouse ACE2-overexpressed cell lines, thus explaining the dramatically increased infectivity of variants carrying these mutations (Fig. 3b). However, the infectivities of other variants were not alerted in ACE2-overexpressed cell lines from other species (Fig. 3a, c–n).

### Neutralization activity of mAbs to the ten variants

**Fig. 4:**
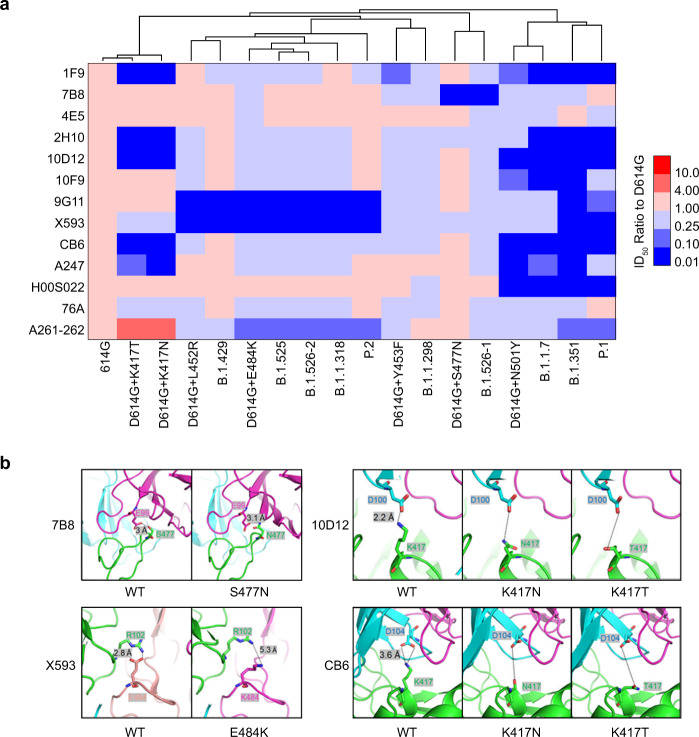
Neutralization analyses of mAbs. **a** Thirteen mAbs were diluted serially and pre-incubated with pseudotyped SARS-CoV-2 variants at 37 °C for 1 h, then added to Huh-7 cell cultures. The heat map shows of mean ID_50_ ratio compared with D614G. The results were obtained from three independent experiments. Red indicates increased neutralization activity and blue indicates decreased neutralization activity, as shown in the scale bar. **b** Structural modelling of the K417N/T, L452R, Y453F, S477N, E484K and N501Y mutations, based on 7chh for X593, RBD-7B8 for 7B8, RBD-Ab1 for 10D 12, and 7c01 for CB6.

To compare the effectivity of neutralizing mAbs against the 10 SARS-CoV-2 variants, the neutralizing activity of 13 mAbs targeting different areas of the receptor-binding domain were tested. As shown in Fig. 4a and Supplementary Figure 2, variants with the highest escape frequencies were B.1.351 and P.1, which escaped from 10 of 13 mAbs (1F9, 2H10, 10D12, 10F9, 9G11, X593, CB6, A247, H00S022, and A261-262). These results were consistent with single-mutation findings involving K417T/N (1F9, 2H10, 10D12, CB6 and A247) and N501Y (1F9, 10D12, 10F9, CB6, A247 and H00S022) and E484K (9G11, X593and A261-262). The variant with the second-highest escape frequency was B.1.1.7, against which seven of 13 mAbs (1F9, 2H10, 10D12, 10F9, CB6, A247 and H00S022) displayed significantly reduced neutralizing activity. Single-mutation analysis indicated that immune escape of the B.1.1.7 variant was mainly caused by the N501Y mutation (Fig. 4a and Supplementary Fig. 3). Similarly, the variants B.1.525, P.2, B.1.526-2 (E484K), B.1.1.318, as well as the E484K single-mutation strain, displayed similar patterns of neutralization sensitivity involving significantly reduced neutralization activity among three mAbs (9G11, X593and A261-262). Furthermore, the variant B.1.429 and L452R single-mutation strain showed reduced susceptibility to mAbs 9G11 and X593; the variant B.1.526-1(S477N) and S477N single-mutation strain showed reduced susceptibility to mAb 7B8, and variant B.1.1.298 and Y453F single-mutation strain showed reduced sensitivity to mAb 1F9. Additionally, the K417N/T mutation caused reduced neutralization activity involving five mAbs (1F9, 2H10, 10D12, CB6, and A247), whereas it increased the neutralization sensitivity of one mAb (A261-262) for more than ten times (Fig. 4a and Supplementary Fig. 3). These results support the body of emergent work^24^ showing that RBD mutations in SARS-CoV-2 variants can affect the neutralization sensitivities of monoclonal antibodies.

### Epitope analysis of the mAbs-RBD complex

To further analyze the reason behind the failure of mAbs to neutralize the tested variants, we performed a structural analysis of the mAbs-RBD complex based on the reported structural information^25–27^. mAb 7B8 binding to the RBD relies on five main hydrogen bonds. The interaction between S477 with E99 is particularly important. The S477N mutation weakens this interaction and causes escape (Fig. 4b). mAb X593 binding to the RBD relies on both salt bridges and hydrogen bonds, particularly the salt bridge E484–R102. This interaction is greatly weakened when the E484K mutation occurs. The contribution of L452 to the affinity between the two proteins is not as significant as that of E484K, causing only minor changes in local structures (Fig. 4b). K417 and D104 located on CDRH3 form salt bridges that play an important role in the process of mAb-CB6 binding to the RBD. K417N/T destroys the salt bridge, thus reducing the affinity significantly. Furthermore, N501Y causes minor changes in the local structure of the CB6-RBD complex, which also weakens the affinity between CDRL and the RBD (Fig. 4b). As for mAb 10D12, K417 and D104 located on CDRH3 form a salt bridge. The K417N/T mutation destroys the salt bridge, reducing affinity significantly. K417N/T and N501Y also cause minor changes in local structure, which further weakens the affinity between CDRL and RBD, especially Y503 on RBD (Fig. 4b). However, the immune escape of 9G11 caused by L452R and E484K cannot be directly explained by salt bridge destruction or hydrogen bond changes. As the two sites are located far from the interaction surface, the mutations causing local conformational changes in the RBD may be the reason behind binding failure.

### Neutralization activities of immunized sera against 10 SARS-COV-2 variants

**Fig. 5:**
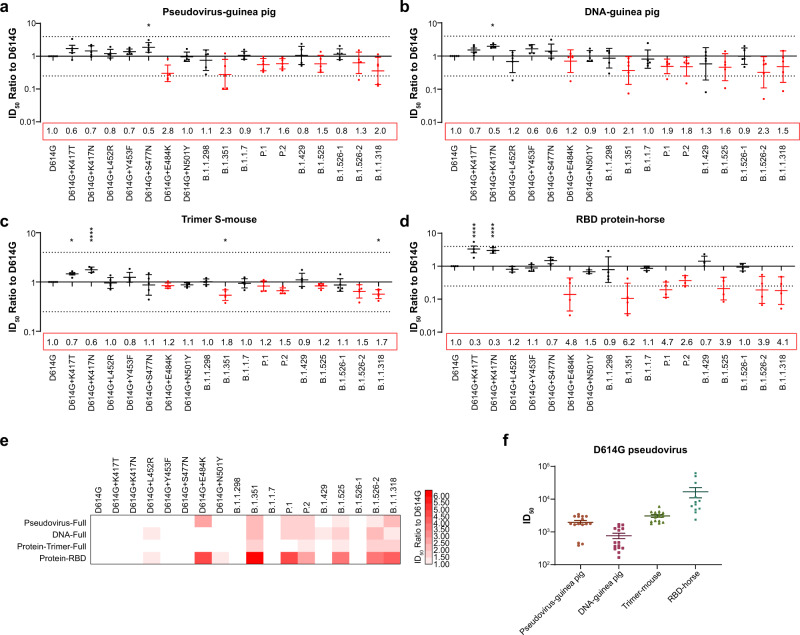
Neutralization analyses of animal immunized sera. Serially diluted serum preparations were incubated with pseudotyped SARS-CoV-2 variants as indicated. The remaining procedure is identical to the procedure in Fig. 5. ID_50_ ratios between the variants and the D614G reference strain are presented. **a**–**d** Dot plots of each serum sample against multiple variants. Data are presented as means ± SEMs. Dashed lines indicate the threshold of fourfold difference. E484K-carrying variants are marked in red. Reduced neutralization, folds compared with the D614G reference strain, is labeled at the bottom of each plot. **e** Heat map of mean ID_50_ ratio, compared with D614G. The shade of red indicates the degree of reduced neutralization (folds), as shown in the scale bar. **f** Neutralization ID_50_ values of different immunized serum samples against pseudotyped D614G virus. All experiments were repeated three times. Asterisks indicate statistical significance.

To determine whether the antigenicity change in the 10 variants could change their neutralizing sensitivity with respect to vaccine immunization, animals were immunized with different types of SARS-CoV-2 antigens: trimer spike protein (in mice), pseudotyped virus (in guinea pigs), recombinant DNA containing full-length spike gene (in guinea pigs) or purified RBD protein (in horses). The neutralization reactions elicited by immunized sera generated with these different antigens were compared among the 10 SARS-CoV-2 variants. Of the 10 variants, only B.1.351, P1, P2, B.1.525, B.1.526-2, and B.1.1.318 displayed obviously reduced sensitivities to immunized sera. Notably, these variants all harbor the E484K mutation (Fig. 5a–e). The reduced sensitivities were observed regardless of SARS-CoV-2 immunogen and source of animal serum (Fig. 5a–e). Furthermore, the neutralization activity against the K417T/N single-mutation strain was increased among all serum samples (Fig. 5e). The neutralization ID_50_ of the various immunogens against the D614G reference strain was shown in Fig. 5f. Regarding the E484K-carrying variants, neutralization sensitivity of the RBD immunized sera reduced 2.6 to 6.2 folds, which is much obvious than other immunogens. On the other hand, the trimer protein immunized sera are much stable, the neutralization activity reduced only 1.1 to 1.8 folds. (Fig. 5a–e). Additionally, although the variants B.1.351 and P.1 carry nearly identical RBD mutations, variant B.1.351 exhibited a much greater reduction in neutralization sensitivity. Moreover, although P.2 only has one additional mutation (V1176F) in the S2 domain, its neutralization activity was less reduced in the pseudovirus- and RBD protein-induced sera compared with the E484K single-mutation strain (Fig. 5a, d).

### Neutralization activities of convalescence sera from patients infected with B.1.1.7 and B.1.351 variants

**Fig. 6:**
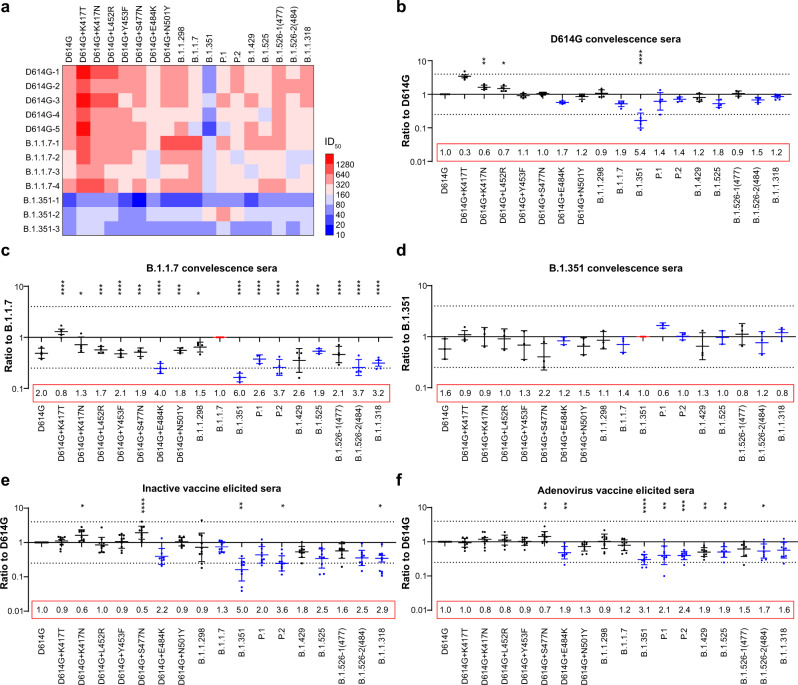
Neutralization analyses of convalescence sera from D614G-, B.1.1.7- and B.1.351- infected patients and vaccine-elicited sera. The procedures were identical to the methods used in animal serum neutralization analyses. **a** Mean neutralization ID_50_ values of different SARS-CoV-2 infected convalescence serum samples against pseudotyped SARS-CoV-2 variants. Red to blue indicates increased neutralization activity high to low as shown in the scale bar. **b** the ID_50_ ratios, compared with the D614G reference strain, are displayed using dot plots. **c** the ID_50_ ratios, compared with the B.1.1.7 variant, are displayed using dot plots. **d** the ID_50_ ratios, compared with the B.1.351 variant, are displayed using dot plots. Data are presented as means ± SEMs. Dashed lines indicate the threshold of fourfold difference. The experiments were repeated twice due to the sample limitation. **e** ID_50_ ratios of sera from inactived-vaccine, compared with the D614G reference strain, are displayed using dot plots. **f** ID_50_ ratios of sera from adenovirus-vaccine, compared with the D614G reference strain, are displayed using dot plots. b-f, data are presented as means ± SEMs. Asterisks indicate statistical significance.

To evaluate the impacts of infection with the B.1.1.7 and B.1.351 variants on neutralization activities, convalescent sera from patients with the two variants and the D614G reference strain were analyzed. The mean neutralizing antibody levels (i.e., ID50 values) were comparable between D614G and B.1.1.7, whereas B.1.351 induced much lower neutralizing antibody production (Fig. 6a). Convalescent sera from D614G infected patients showed a neutralization pattern similar to the pattern exhibited by SARS-CoV-2 immunized animal sera (Fig. 6b). However, the B.1.351 variant-infected sera showed better neutralization activity against P.1, the variant itself and E484K-carrying variants, relative to the D614G reference strain (Fig. 6c). B.1.1.7 variant-infected sera also showed the highest neutralization activity against the variant itself; it was comparatively resistant to B.1.351 and other E484K-carrying sera (Fig. 6d).

### Neutralization activities of vaccine elicited sera

Neutralization ability of sera elicited by two vaccines approved in China was tested, including inactivated vaccine KCONVAC^28^ (Shenzhen Kangtai Biological Products Co.) and adenovirus vaccine Ad5-nCoV^29^ (CanSino Biologics Inc.). The results were similar to those observed in the immunized animal sera and D614G convalescent patient sera experiments. However, an obvious decrease in neutralization sensitivity of E484K-containing SARS-CoV-2 variants was apparent in the vaccine group, especially the inactivated vaccine group (Fig. 6e and f) compared to the convalescence sera group (Fig. 6b). The decrease in neutralization against P.1 was also less pronounced compared with B.1.351; however, the increase in neutralization against the K417T/N single-mutation strain was less than that of the immunized animal sera or convalescence sera group (Fig. 6e and f). Upon comparison of the two vaccines, the results were quite similar because they both targeted full-length spike protein. Overall, although the variants reduced the neutralization abilities of vaccines by approximately 0.9–5.0-fold for the inactive vaccine-elicited sera and 0.9–3.1-fold for the adenovirus vaccine-elicited sera, both vaccines continued to exhibit a protective effect (Fig. 6e and f).

## Discussion

Recently, emerging SARS-CoV-2 variants have attracted the attention of scientists worldwide. After the onset of the SARS-CoV-2 outbreak, the D614G strain rapidly replaced the original virus strain and became the dominant variant^3^. Further studies showed that the host cell infectivity of D614G was increased, compared with the original virus, because of mutation-related structure changes^21,30^. Subsequently, many SARS-CoV-2 variants were reported to be spread rapidly in various countries. The World Health Organization and the United States CDC have reported several variants of interest (VOIs), which are associated with established or suspected phenotypic implications and have been caused by community transmission or been detected in multiple countries. As of March 30, 2021, there were nine VOIs, of which three were variants of concern (VOCs; e.g., B.1.1.7, B.1.351 and P.1.) that showed increased transmissibility and potentially reduced neutralization by convalescent and post-vaccination sera^6^. Furthermore, a previous study demonstrated increased binding of N501Y to mouse ACE2, which implies differences in host tropism^31^. Here, we evaluated the host tropism characteristics of 10 SARS-CoV-2 variants, which variants included most VOIs and additional potentially important variants. Pseudoviruses of multiple variants and single mutants at RBD sites presumably related to these variants were examined for their ability to infect four SARS-CoV-2 susceptible cells. Slightly enhancements of infectivity were observed among most of the tested variants, compared with the D614G reference strain, whereas the B.1.1.298 variants (mink cluster 5) displayed significantly decreased infectivity. Dr Fomsgaard’s study using authentic virus showed that the mink cluster 5 was less pronounced and had an approximate 10-fold lower titer 24 h post-inoculation compared to human SARS-CoV-2 isolates^32^, which was also consistent with our previous study focused on mink SARS-CoV-2 variants^33^. Further analysis showed that M1229I may be the key mutation that responsible for the reduced infectivity. Moreover, the increased infectivity of L452R mutation and B.1.429 variant were consistent with the reports by Deng et al.^34^. However, our results are not as obvious as theirs (2.7-folds vs. 5.8–27.5-folds) as infected the same 293T-ACE2-TMPRSS2 cells, which may be due to the different sensitivity of the pseudovirus systems. The RLU signals in our VSV-based SARS-CoV-2 pseudovirus are around 10^6^–10^7^, whereas the RLU signals of their HIV-based SARS-CoV-2 pseudovirus are about 10^4^-10^5^. In the VSV-based system, when we decreased the concentration of the virus and harvested the signal at around 10^4^–10^5^, it was not stable enough to yield reliable results. In addition, we also found slight enhancements of infectivity involving the B.1.351, B.1.525, B.1.526, and B.1.1.318 variants, suggesting that these variants should receive close attention.

Furin, TMPRSS2 and Cathepsin L are important proteolytic enzymes, which are key regulators of SARS-CoV-2 infection^22,23^. Overexpression of these enzymes facilitates cellular infection. Surprisingly, almost all SARS-CoV-2 variants further enhanced the enzymes that promoted SARS-CoV-2 infection, especially when furin was overexpressed in 293T-ACE2 cells. When TMPRSS2 overexpressed infection was compared to mock 293T-ACE2 cells, the enhancement of infectivity by TMPRSS2 for the P.1 variant was almost fourfold that for the D614G variant. The increased affinity to ACE2 receptors caused by RBD mutations (e.g., E484K, N501Y)^31^ or increased cleavage activity by mutations adjusted to the furin site (e.g., P681H)^35^ may facilitate the function of enzymes. The underlying mechanisms of these increased infectivities require further analyses. The underlying mechanisms of these increased infectivities require further analyses.

Cross-species infections caused by viral mutations contribute to the extensive spreading of many animal-derived viruses in human populations. In this study, we evaluated the abilities of current SARS-CoV-2 variants to bind ACE2 proteins of 14 different animal species. We found that K417N/T, E484K, and N501Y mutations significantly increased the ability of SARS-CoV-2 to infect 293 T cells overexpressing mouse ACE2; variants carrying these mutations (i.e., B.1.1.7, B.1.351, P.1, P.2, B.1.429, B.1.525, B.1.526-2, and B.1.1.318) showed similar changes in infectivity. These results suggest the need for careful monitoring of new variants in mice, which may lead to further virus mutations and prolong the spread of the disease.

Regarding neutralization, we found that mutations in the RBD enabled to escape from various mAbs; these escape findings were consistent with the activities of SARS-CoV-2 variants carrying the corresponding mutations. Six antibodies showed reduced neutralization against N501Y, including mAbs CB6, which was consistent with previous reports^36–38^. Chen et al. also examined a group of monoclonal antibodies, but did not find out significantly reduced neutralization, which may be due to the different groups of monoclonal antibodies that they tested^39^. Variants with more mutations in the RBD region (e.g., B.1.1.7, B.1.351, and P.1) more frequently escaped from mAbs. The results indicate that the neutralization activities of mAbs against epidemic variants should be examined during the development of new therapeutic mAbs; additionally, specific mAbs are presumably more effective against specific variants, implying that cocktail therapy might be appropriate in clinical practice.

Analysis of serum neutralization resistance revealed that most single mutations, including those in the RBD region, could not directly and substantially change the serum neutralization effect on SARS-CoV-2. However, variants carrying the E484K mutation had distinct reductions in neutralization susceptibility. Moreover, some studies have shown that selective pressure from therapeutic mAbs or convalescent serum could induce E484K or E484Q mutations^24,40^. These results indicate the importance of E484 in the viral epitope. Our previous study revealed that the K417N mutation in the RBD region of B.1.351 led to the enhanced convalescence sera neutralization activity^10^. The increased neutralization against K417N by antibodies against RBM was also observed in other group^39^. Here we found that the K417T mutation in the P.1 variant has a similar effect. The increase in K417N sensitivity to serum neutralization has been discussed in our previous paper^10^. K417 forms hydrogen bonds with N370 of S protein resulting in stabilized and closed conformation, which presents a reduced overall area accessible to antibodies or ACE2^41^. The K417N/T mutation increases the probability of conversion to the open conformation, thus exposing epitopes to neutralizing antibodies, which would increase the likelihood of virus neutralization by sera containing polyclonal antibodies. Although variants B.1.351 and P.1 have similar RBD mutation sites, the neutralization resistance of P.1 was less robust than the resistance of B.1.351. Mutations in other regions of the virus (e.g., NTD or S2) may also cause antigenicity changes^42^. Furthermore, the findings that neutralization by full-length trimer spike-immunized sera was reduced to a lesser extent than RBD-immunized sera suggest that full-length spike protein may induce more complete antibodies. Our preliminary study found that mice immunized with the NTD fragment produced almost no neutralizing antibody, while mice immunized with S2 fragment produced neutralizing antibody. Because the S2 region is a key region that mediates virus fusion, mutation of this region may change viral structure and influence neutralization activity. These results emphasize the need to consider regions other than the RBD in vaccine design efforts. Notably, the RBD is a robust immunogen for neutralizing antibody production, and the corresponding antibody titer is also relatively high. However, modification of E484K in the RBD led to the most marked change in immune escape. Therefore, the RBD alone may not be an ideal immunogen for vaccine development.

Neutralization ability of sera elicited from two vaccines approved in China were tested. E484K was found to be the key mutation that caused the most obvious neutralization insensitivity, while B.1.351 was the variant that exhibited the most significant immune escaped. Because the B.1.351 variant can escape from a large number of mAbs and exhibits resistance to most vaccine-induced protection^10,11^, the B.1.351 variant has been regarded as an important candidate for new generations of vaccines. However, neutralization analyses of convalescent sera from D614G-, B.1.1.7- and B.1.351-infected patients indicate that B.1.351 variant induced much lower antibody production, compared with other variants. There were only three patients in our tests, more samples are needed to further analyze the immunogenicity of B.1.351.

In summary, we systematically analyzed the infectivity and host tropism of 10 SARS-CoV-2 variants. The infectivities of most tested SARS-CoV-2 variants were slightly increased compared with the reference strain, and also exhibited considerable enhancements of infectivity in the presence of furin, TMPRSS2, and cathepsin L. Our results demonstrate that the K417N/T, E484K, and N501Y mutations change host tropism, implying possible transmission of SARS-CoV-2 variants in mice. The neutralization activity of mAbs, immunized sera and convalescent sera from different variants infected patients were analyzed against the 10 variants, which would provide insights for the development of therapeutic antibodies and vaccine design.

## Methods

### Cells

Three human cell lines Huh-7, Calu-3, and 293 T was from the Japanese Collection of Research Bioresources (Cat: 0403) and American Type Culture Collection (ATCC, Cat: HTB-55 and CRL-3216). Two monkey cell lines LLC-MK2 and Vero were from ATCC (Cat: CCL-7 and CCL-81). 293T-hACE2, 293T-hACE2-Furin, 293T-hACE2-TMPRSS2 and 293T-hACE2-Cathepsin L overexpressing cells were human ACE2, Furin, TMPRSS2, and Cathepsin L-stably expressing 293 T cells. Receptor-transiently overexpressing cells were prepared by transfecting 293 T cells with plasmids containing ACE2 from different species. Cells were cultured using Dulbecco’s modified Eagle medium (DMEM, high glucose; Hyclone) supplied with 100 U/mL of Penicillin-Streptomycin solution (Gibco), 20mM N-2-hydroxyethylpiperazine-N-2-ethane sulfonic acid (HEPES, Gibco) and 10% fetal bovine serum (FBS, Pansera ES, PAN-Biotech), in a 5% CO_2_ environment at 37 °C; cells were passaged at intervals of 2–3 days using 0.25% Trypsin-EDTA (Gibco). Lipofectamine 2000 (Invitrogen) was used as a transfection reagent. For a T75 culture flask, 30 μg plasmid and 30 μL Lipofectamine 2000 reagent was diluted with 0.75 mL opti-MEM (Gibco) respectively. After 5 minutes, the diluted plasmid was mixed with diluted Lipofectamine 2000 reagent. The mixture was stored for another 15 min and added into the cell culture medium. The cell culture medium was changed 4–6 h after transfection. All cells were used 24-48 h after transfection.

### Monoclonal antibodies

mAbs A261-262, A247 and 76 A (acquired from Professor Linqi Zhang of Tsinghua University, Beijing, China) were produced by RBD-specific single B cells isolated by FACS from SARS-CoV-2 Wuhan-1 strain-infected patients. mAb H00S022 (Sino Biological, Beijing, China) was screened from a phage display scFv library constructed from splenic mRNA of mice immunized with recombinant SARS-CoV-2 RBD of Wuhan-1 strain. mAbs 1F9, 7B8, 4E5, 2H10, 10D12, 10F9 and 9G11 (Biocytogen Pharmaceuticals [Beijing] Co., Bejing, China) were produced by humanized mouse hybridoma against the RBD of SARS-CoV-2 Wuhan-1 strain. X593 (acquired from Prof. Sunney Xie of Peking University, Beijing, China) was identified by high-throughput single-cell RNA and VDJ sequencing of antigen-enriched B cells from 60 convalescent patients. mAb CB6(acquired from Prof. Jinghua Yan, Institute of Microbiology, Chinese Academy of Sciences, Beijing, China) was generated by FACS sorting of the membrane B cells from a patient convalescing from COVID-19 and clone the V_H_ and V_L_ genes to human IgG1.

### Immunized sera

The study protocol was approved by the Animal Care and Use Committee at the National Institutes for Food and Drug Control (NIFDC). The Animals were handled in accordance with the protocol and guidelines for laboratory animal care and use. Mice were immunized with purified Trimer protein with aluminum adjuvant (20 µg per mouse, once per week for 3 weeks). Serum samples were collected at 4 weeks after the third immunization. Serum samples from 10 mice of each group were pooled to produce combined samples. Each two mice were combined to make one sample. Guinea pigs were immunized with SARS-CoV-2-Spike plasmid at 200 µg per guinea pig or peudotyped virus at 6×10^5^ TCID_50_ per guinea pig(once every 2 weeks for 6 weeks). Serum samples from five guinea pigs in each group were collected 28 days after the third immunization. Horses were immunized with SARS-CoV-2 RBD protein plus Freund’s incomplete adjuvant (once every 10 days for 30 days) at doses of 3 mg, 6 mg and 12 mg. Serum samples were collected at 1 week after the third immunization.

### Convalescence sera

Twenty convalescence serum samples were collected from patients with COVID-19, 2-3 months after SARS-CoV-2 infection. Of these 10 samples, 5 were from D614G reference strain-infected patients, four were from B.1.1.7-infected patients, and three were from B.1.351-infected patients. All patients provided written informed consent to participate in the study.

### Sera from vaccinated participants

Ten serum samples from individuals immunized with the inactivated vaccine (KCONVAC, Shenzhen Kangtai Biological Products Co.) were collected at 14 days after completion of a three-dose immunization procedure (5 µg/dose; doses given at 0, 28, and 58 days). The study protocol was approved by the Ethics Committee of Jiangsu Provincial Center for Disease Control and Prevention. Written informed consent was signed by all volunteers prior to blood collection.

Ten serum samples were collected from individuals immunized with the adenovirus vaccine (Ad5-nCoV, CanSino Biologics Inc.) at 28 days after a standard immunization procedure (one dose at day 0; 300 µl/dose). The study protocol was approved by the Ethics Committee of Jiangsu Provincial Center for Disease Control and Prevention. Written informed consent was obtained from all volunteers prior to blood collection.

### SARS-CoV-2 pseudotyped virus

In accordance with our published method^21^, the spike protein expression plasmid was constructed using GenBank sequence MN908947. The spike gene was codon-optimized and inserted into pcDNA3.1 plasmid between the enzyme digestion sites BamHI and XhoI. Replication-defective SARS-CoV-2 viral particles were generated by transfection with pcDNA3.1- SARS-CoV-2 and concurrent infection with G*ΔG-VSV (Kerafast). The cell supernatant containing pseudotyped virus was harvest 24 and 48 h later, then aliquoted and stored at −80 °C. Site-directed mutagenesis based on circular PCR was used to construct mutants of SARS-CoV-2 pseudotyped virus. DpnI (NEB) was used to digest template DNA. Primers used for mutation are listed in Supplementary Table 1. Pseudotyped SARS-CoV-2 variants were quantified via RT-PCR using VSV P protein as an internal control. Viruses of multiple variants were diluted to the same number of copies before use in experiments.

### Infection and neutralization assays

As described in our previous paper^2^, pseudotyped viruses were purified through 25% sucrose cushion by ultra-centrifugation. 7 mL pseudovirus was loaded on 3 mL sucrose cushion, and centrifuged at 10,0000 g for 4 hours at 4 °C. The RNA of the sediment was extracted and quantified by real-time PCR. The same copy numbers of different pseudotyped SARS-CoV-2 variants were mixed with the indicated cells for infection assays. For neutralization assays, the pseudoviruses were diluted to make sure the control group (without antibody) infected Huh7 cells at the same level (same RLU after 24 h infection, Supplementary Figure 4). mAbs or sera were serially diluted and preincubated with the pseudotyped virus at 37 °C for 1 h, then mixed with Huh-7 cells. The cells were then incubated for 20-28 hours at 37 °C, in an environment containing 5% CO_2_. Chemiluminescence signals were collected by PerkinElmer Ensight using luciferase substrate (PerkinElmer). Duplicated wells were analyzed for each group. Each experiment was repeated for 2–5 times. Reed-Muench method was used to calculate the half-maximal inhibition dilution (ID_50_)^43^.

### Structural modelling

The spike protein was modelled based on the following Protein Data Bank coordinate sets: RBD-7B8 for 7B8, RBD-Ab5 for 9G11, RBD-Ab1 for 10D 12, 7chh for X593 and 7c01 for CB6. Mutant simulations were carried out in the Mutabind2 web server. MutaBind2 calculates changes in binding affinity upon single or multiple mutations and provides a structural model of the mutated complex. The MutaBind2 model uses molecular mechanics force fields, statistical potentials and fast side-chain optimization algorithms built via a random forest method. Protein-protein interactions were calculated in PDBePISA. Images demonstrating structures were generated in PyMOL.

### FACS analysis

Cell surface expression of the spike protein was assessed by flow cytometry. 293 T cells were transfected using the same procedure as for packaging the pseudotyped virus (see above). The medium was removed after transfection for 36 h, following which the cells were digested to produce a single-cell suspension, washed once with PBS, and resuspended with PBS solution containing 1% BSA at 1 × 10^6^ cells/tube. MW06 anti-spike antibody (Kohnoor Science & Technology Co., Beijing, China, no neutralization escape for all the tested variants) solution with a final concentration of 1 μg/mL was used as the primary antibody and a FITC-labeled goat anti-human IgG (ZF-0308, Zhongshan Jinqiao, Beijing, China) with a final concentration of 6 μg/mL was added as the secondary antibody. The fluorescent signal was examined using a BD FACS CantoTM II Flow Cytometer (BD, Franklin Lakes, NJ).

### Statistics and reproducibility

ID_50_ was calculated using the Reed-Muench method. Graphical representations were generated using GraphPad Prism 8. One-way ANOVA and Holm-Sidak’s multiple comparisons test were used to identify differences relative to the D614G reference strain. Values are shown as means ± SEMs. For all figures, *P < 0.05, **P < 0.01, ***P < 0.005, and ****P < 0.001.

### Reporting summary

Further information on research design is available in the Nature Research Reporting Summary linked to this article.

## Supplementary information

Transparent Peer Review File

Supplementary Information

Reporting Summary

## Data Availability

The raw data have been deposited in Figshare (https://figshare.com/articles/figure/Comparison_of_10_emerging_SARS-CoV-2_Variants_infectivity_animal_tropism_and_antibody_neutralization/14526894). Other data are available from the authors upon reasonable request.

## References

[1] Garcia-Beltran WF (2021). Multiple SARS-CoV-2 variants escape neutralization by vaccine-induced humoral immunity. Cell.

[2] Li Q (2020). The impact of mutations in SARS-CoV-2 spike on viral infectivity and antigenicity. Cell.

[3] Korber B (2020). Tracking changes in SARS-CoV-2 Spike: evidence that D614G increases infectivity of the COVID-19 virus. Cell.

[4] Oude Munnink BB (2021). Transmission of SARS-CoV-2 on mink farms between humans and mink and back to humans. Science.

[5] Koopmans M (2021). SARS-CoV-2 and the human-animal interface: outbreaks on mink farms. Lancet Infect. Dis..

[6] WHO. Weekly epidemiological update on COVID-19 - 13 April 2021. https://www.who.int/publications/m/item/weekly-epidemiological-update-on-covid-19---13-april-2021.

[7] Davies, N. G. et al. Estimated transmissibility and impact of SARS-CoV-2 lineage B.1.1.7 in England. *Science* **372**, 10.1126/science.abg3055 (2021). 10.1126/science.abg3055 PMC8128288 33658326

[8] Graham, M. S. et al. Changes in symptomatology, reinfection, and transmissibility associated with the SARS-CoV-2 variant B.1.1.7: an ecological study. *Lancet Public Health*, 10.1016/S2468-2667(21)00055-4 (2021). 10.1016/S2468-2667(21)00055-4 PMC8041365 33857453

[9] Shen X (2021). SARS-CoV-2 variant B.1.1.7 is susceptible to neutralizing antibodies elicited by ancestral spike vaccines. Cell Host Microbe.

[10] Li, Q. et al. SARS-CoV-2 501Y.V2 variants lack higher infectivity but do have immune escape. *Cell*, 10.1016/j.cell.2021.02.042 (2021). 10.1016/j.cell.2021.02.042 PMC7901273 33735608

[11] Zhou, D. et al. Evidence of escape of SARS-CoV-2 variant B.1.351 from natural and vaccine-induced sera. *Cell*, 10.1016/j.cell.2021.02.037 (2021). 10.1016/j.cell.2021.02.037 PMC7901269 33730597

[12] Sabino EC (2021). Resurgence of COVID-19 in Manaus, Brazil, despite high seroprevalence. Lancet.

[13] Naveca, F., Nascimento, V., Souza, V., Corado, A. & Bello, G. COVID-19 epidemic in the Brazilian state of Amazonas was driven by long-term persistence of endemic SARS-CoV-2 lineages and the recent emergence of the new Variant of Concern P.1. (2021).

[14] Shen, X. et al. Neutralization of SARS-CoV-2 Variants B.1.429 and B.1.351. *The New England journal of medicine*, 10.1056/NEJMc2103740 (2021). 10.1056/NEJMc2103740 PMC8063884 33826819

[15] Zhou, H. et al. B.1.526 SARS-CoV-2 variants identified in New York City are neutralized by vaccine-elicited and therapeutic monoclonal antibodies. *bioRxiv*, 10.1101/2021.03.24.436620 (2021). 10.1128/mBio.01386-21 PMC8406170 34311587

[16] McCallum, M. et al. SARS-CoV-2 immune evasion by variant B.1.427/B.1.429. *bioRxiv*, 10.1101/2021.03.31.437925 (2021). 10.1126/science.abi7994 PMC9835956 34210893

[17] Annavajhala, M. K. et al. A Novel SARS-CoV-2 Variant of Concern, B.1.526, Identified in New York. *medRxiv: the preprint server for health sciences*, 10.1101/2021.02.23.21252259 (2021).

[18] Public, Health & England. SARS-CoV-2 variants of concern and variants under investigation in England. https://www.gov.uk/government/publications/investigation-of-novel-sars-cov-2-variant-variant-of-concern-20201201 (2021).

[19] Hogan, C. A. et al. Optimizing SARS-CoV-2 Variant of Concern Screening: Experience from British Columbia, Canada, Early 2021. 2021.2003.2023.21253520, 10.1101/2021.03.23.21253520 %J medRxiv (2021).

[20] Ikegame, S. et al. Qualitatively distinct modes of Sputnik V vaccine-neutralization escape by SARS-CoV-2 Spike variants. 2021.2003.2031.21254660, 10.1101/2021.03.31.21254660 %J medRxiv (2021).

[21] Bestle, D. et al. TMPRSS2 and furin are both essential for proteolytic activation of SARS-CoV-2 in human airway cells. *Life Sci Alliance* **3**, 10.26508/lsa.202000786 (2020). 10.26508/lsa.202000786 PMC7383062 32703818

[22] Zhao MM (2021). Cathepsin L plays a key role in SARS-CoV-2 infection in humans and humanized mice and is a promising target for new drug development. Signal Transduct. Target Ther..

[23] Hoffmann M (2020). SARS-CoV-2 cell entry depends on ACE2 and TMPRSS2 and is blocked by a clinically proven protease inhibitor. Cell.

[24] Liu Z (2021). Identification of SARS-CoV-2 spike mutations that attenuate monoclonal and serum antibody neutralization. Cell Host Microbe.

[25] Wang et al. Three epitope-distinct human antibodies from RenMab mice neutralize SARS-CoV-2 and cooperatively minimize the escape of mutants. *Cell Discov*, (2021). 10.1038/s41421-021-00292-z PMC8290868 34285195

[26] Cao Y (2020). Potent neutralizing antibodies against SARS-CoV-2 identified by high-throughput single-cell sequencing of convalescent patients’ B cells. Cell.

[27] Shi R (2020). A human neutralizing antibody targets the receptor-binding site of SARS-CoV-2. Nature.

[28] Pan HX (2021). Immunogenicity and safety of a severe acute respiratory syndrome coronavirus 2 inactivated vaccine in healthy adults: randomized, double-blind, and placebo-controlled phase 1 and phase 2 clinical trials. Chin. Med. J..

[29] Wu S (2020). A single dose of an adenovirus-vectored vaccine provides protection against SARS-CoV-2 challenge. Nat. Commun..

[30] Mansbach, R. A. et al. The SARS-CoV-2 Spike Variant D614G favors an open conformational state. *bioRxiv*, 10.1101/2020.07.26.219741 (2020). 10.1126/sciadv.abf3671 PMC8051874 33863729

[31] Starr TN (2020). Deep mutational scanning of SARS-CoV-2 receptor binding domain reveals constraints on folding and ACE2 binding. Cell.

[32] Lassaunière, R. et al. SARS-CoV-2 spike mutations arising in Danish mink and their spread to humans. https://files.ssi.dk/Mink-cluster-5-short-report_AFO2 (2020).

[33] Zhang L (2021). Cellular tropism and antigenicity of mink-derived SARS-CoV-2 variants. Signal Transduct. Target Ther..

[34] Deng, X. et al. Transmission, infectivity, and neutralization of a spike L452R SARS-CoV-2 variant. *Cell*, 10.1016/j.cell.2021.04.025 (2021). 10.1016/j.cell.2021.04.025 PMC8057738 33991487

[35] Lubinski, B., Tang, T., Daniel, S., Jaimes, J. A. & Whittaker, G. R. Functional evaluation of proteolytic activation for the SARS-CoV-2 variant B.1.1.7: role of the P681H mutation. *bioRxiv*, 10.1101/2021.04.06.438731 (2021). 10.1016/j.isci.2021.103589 PMC8662955 34909610

[36] Cheng L (2021). Impact of the N501Y substitution of SARS-CoV-2 Spike on neutralizing monoclonal antibodies targeting diverse epitopes. Virol. J..

[37] Zhou D (2021). Evidence of escape of SARS-CoV-2 variant B.1.351 from natural and vaccine-induced sera. Cell.

[38] Cao, Y. et al. Humoral immune response to circulating SARS-CoV-2 variants elicited by inactivated and RBD-subunit vaccines. *Cell research*, 1–10, 10.1038/s41422-021-00514-9 (2021). 10.1038/s41422-021-00514-9 PMC8138844 34021265

[39] Chen RE (2021). Resistance of SARS-CoV-2 variants to neutralization by monoclonal and serum-derived polyclonal antibodies. Nat. Med..

[40] Truong, T. T. et al. Persistent SARS-CoV-2 infection and increasing viral variants in children and young adults with impaired humoral immunity. *medRxiv: the preprint server for health sciences*, 10.1101/2021.02.27.21252099 (2021). 10.1016/j.ebiom.2021.103355 PMC8072072 33915337

[41] Walls AC (2020). Structure, Function, and Antigenicity of the SARS-CoV-2 Spike Glycoprotein. Cell.

[42] McCallum, M. et al. N-terminal domain antigenic mapping reveals a site of vulnerability for SARS-CoV-2. *bioRxiv*, 10.1101/2021.01.14.426475 (2021). 10.1016/j.cell.2021.03.028 PMC7962585 33761326

[43] Nie J (2020). Quantification of SARS-CoV-2 neutralizing antibody by a pseudotyped virus-based assay. Nat. Protoc..

