# Supplementary material for: Ten emerging SARS-CoV-2 spike variants exhibit variable infectivity, animal tropism, and antibody neutralization
Source: Commun Biol. 2021 Oct 13;4:1196. doi: 10.1038/s42003-021-02728-4 (PMC8514557; doi:10.1038/s42003-021-02728-4)
Supplement: Supplementary file 2 — Supplementary Information [file 42003_2021_2728_MOESM2_ESM.pdf]

Mutation	Oligonucleotides
L5F	ATCCGCCACCATGTTCGTGTTCTTCGTGCTGCTGCCTCTGGTGAGC
S13I	CTGCCTCTGGTGAGCATCCAGTGCGTGAATCTG
L18F	TGCGTGAATTTTACCACCAGAACCCAGCT
L18F, T20N, P26S	TGCGTGAATTTTACCAACAGAACCCAGCTGCCTTCTGCCTACACC
Q52R	AGTAGTGTATTACATAGTACCCGGGACCTGTTCCTACCTTTCTTCAG
HV69-70Del	CGTGACCTGGTTCCACGCCATCAGCGGCACCAATGGCACCAAGAGATTC
A67V-HV69-70Del	TTCAGTAACGTGACCTGGTTCCACGTCATCAGCGGCACCAATGGCA
D80A	CAATGGCACCAAGAGATTCGCCAATCCTGTGCTGCCTTTCAAT
T95I	CGGCGTGTACTTCGCCAGCATCGAGAAGAGCAATATCATCA
D138Y	TTCCAGTTCTGCAATTACCCTTTCCTGGGTGTT
Y144/145Del	AATGACCCTTTCCTGGGTGTTTATCATAAGAACAACAAGAGC
W152C	CATAAGAACAACAAGAGCTGCATGGAGAGCGAGTTCCG
R190S	TTTCAAGAATCTGAGCGAGTTCGTGTTCA
D215G	CACCCATTAATCTGGTGAGAGGCCTGCCTCAGGGCTTCAGC
LAL242-244Del	TATCACCAGATTCCAGACCCTGCACAGATCATATCTTACACC
D253G	GATCATATCTTACACCAGGCGGTTCGTCAAGCGGTTGGACCGC
K417T	CAGGGCAGACCGGCACGATCGCCGACTACAAT
K417N	CGCCAGGGCAGACCGGCAATATCGCCGACTACAATTAC
L452R	TTGGAGGCAATTACAATTACCGGTACAGACTGTTCAGAAAGAG
Y453F	GTTGGAGGCAATTACAATTACCTGTTCAGACTGTTCAGAAAGAGCAATC
S477N	GCACCGAGATCTACCAGGCCGGCaacACACCGTGTAATGGCGTGGAGGGC
E484K	CACCGTGTAATGGCGTGAAGGGCTTCAATTGCTACTTCC
N501Y	AGAGCTACGGCTTCCAGCCTACCtacGGCGTGGGCTACCAGCCTTACAG
A570D	CAACAATTCGGCAGAGACATCGACGACACCACAGATGCTGTAAGAGAC
D614G	GTGGCCGTGCTGTACCAGGGCGTGAATTGCACCGAGGT
H655Y	CTGCCTGATCGGCGCCGAGTACGTGAATAATAGCTACG
Q677H	GCCAGCTACCAGACCCATACCAATAGCCCTAGA
P681H	CTACCAGACCCAGACCAATAGCCACAGAAGAGCCAGAAGCGTGGCCAGCC
A701V	CTACACCATGAGCCTGGGCGTGGAGAATAGCGTGGCCTAC
I692V	CAGAAGCGTGGCCAGCCAGAGCGTGATCGCCTACACCATGAGCCTG
T716I	CAATAATAGCATCGCCATCCCTATCAATTTCACCATCAGCGTGACCAC
D796H	ACAAGACTCCGCCGATCAAGCACTTCGGCGGCTTCAATTTCA
F888L	CACATCGGGCTGGACTTTAGGCGCCGGAGCAGCGTTG
Q957R	CGTGGTGAATCAGAATGCCCGGGCCCTGAATACCCTGGT
S982A	TACTCAACGACATCCTGGCGAGACTGGACAAGGTGGAGGCCGA
T1027I	CAATCTGGCCGCCATCAAGATGAGCGAGTGC
D1118H	ACGAGCCTCAGATCATCACCACCcacAATACCTTCGTGAGCGGCAA
V1176F	GGGAATCAATGCCAGCTTTGTGAATATCCAGAAGGAAAT
M1229I	CATCGCCGGCCTGATCGCCATCGTGATCGTGACCATCATGCTGTGCTGCA

Supplementary Table 1 Primers to construct SARS-CoV-2 variants.

Forward primers for site-directed mutagenesis are listed. The reverse primers are reverse complement of the forward primers.

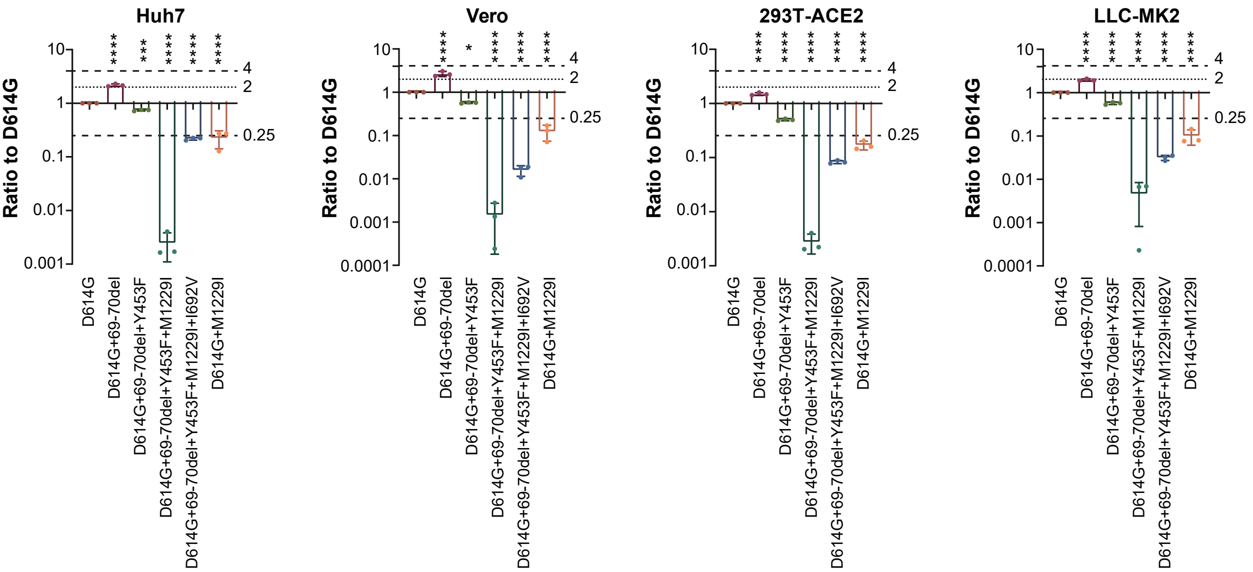

Supplementary Figure 1 Analysis of B.1.1.298, related to Figure 2.
SARS-CoV-2-susceptible cells as indicated were used to compare the infectivities of indicated mutations that composed B.1.1.298. The method was the same as Figure 2. Data are presented as means±SEMs from three independent experiments.

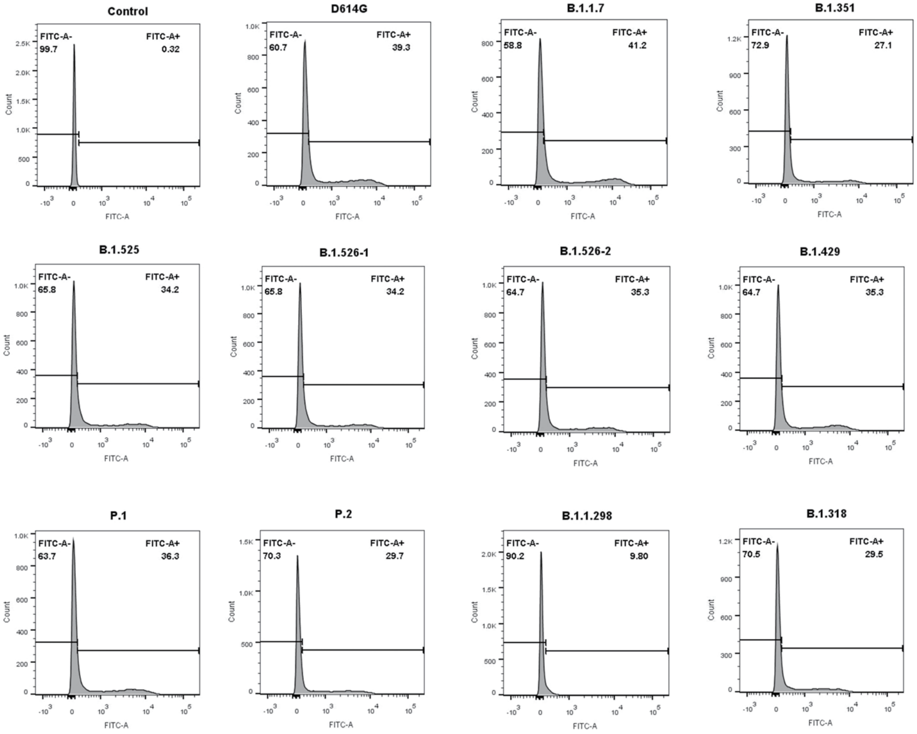

Supplementary Figure 2 FACS analysis of membrane expression of spike protein.

Same amount of spike protein expression plasmids were transfected to 293T cells. The membrane expression level of spike protein was detected by FACS. One of three representative experiments was shown.

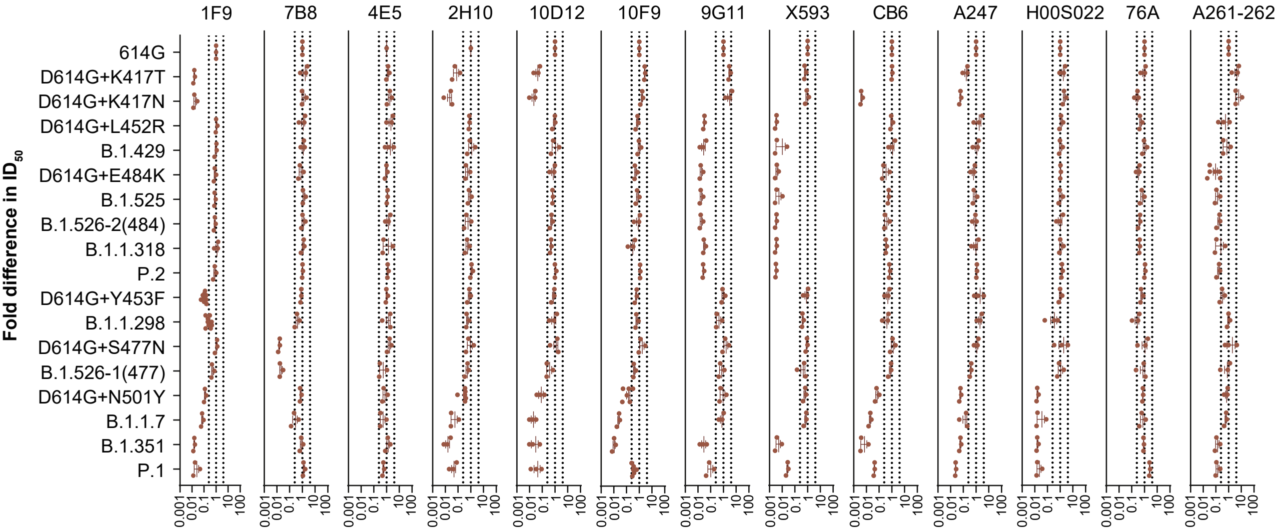

Supplementary Figure 3 Neutralization analyses of mAbs, related to Figure 4a.

Thirteen mAbs were examined for neutralization against ten SARS-CoV-2 variants. ID50 ratios compared with D614G are presented as means±SEMs. Dashed lines indicate the threshold of fourfold difference. All experiments were repeated at least three times.

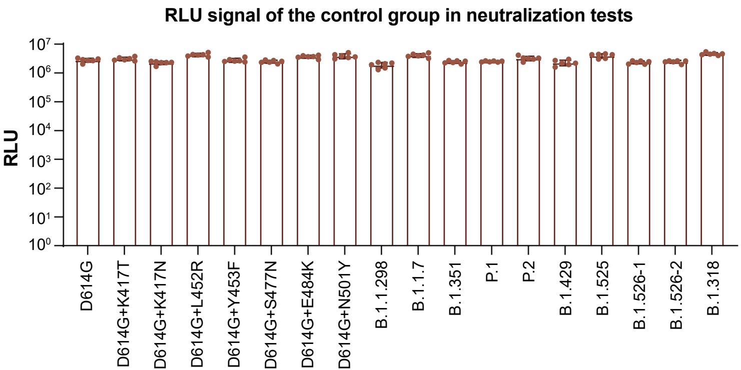

Supplementary Figure 4 The infectivity of pseudoviruses used in the neutralization experiment. Pseudovirus of different variants were diluted to make sure the same level of infectivity before neutralization experiments were performed. Data are presented as means±SEMs. One representative experiment was shown. Six duplicated data was collected for each group.
