# Supplementary material for: Ten emerging SARS-CoV-2 spike variants exhibit variable infectivity, animal tropism, and antibody neutralization
Source: Commun Biol. 2021 Oct 13;4:1196. doi: 10.1038/s42003-021-02728-4 (PMC8514557; doi:10.1038/s42003-021-02728-4)
Supplement: Supplementary file 3 — Reporting Summary [file 42003_2021_2728_MOESM3_ESM.pdf]

nature portfolio

Corresponding author(s): Youchun Wang

Last updated by author(s): Sep 3, 2021

Reporting Summary

Nature Portfolio wishes to improve the reproducibility of the work that we publish. This form provides structure for consistency and transparency in reporting. For further information on Nature Portfolio policies, see our Editorial Policies and the Editorial Policy Checklist.

Statistics

For all statistical analyses, confirm that the following items are present in the figure legend, table legend, main text, or Methods section.

n/a	Confirmed	
☐	☒	The exact sample size (*n*) for each experimental group/condition, given as a discrete number and unit of measurement
☐	☒	A statement on whether measurements were taken from distinct samples or whether the same sample was measured repeatedly
☐	☒	The statistical test(s) used AND whether they are one- or two-sided<br>*Only common tests should be described solely by name; describe more complex techniques in the Methods section.*
☒	☐	A description of all covariates tested
☐	☒	A description of any assumptions or corrections, such as tests of normality and adjustment for multiple comparisons
☐	☒	A full description of the statistical parameters including central tendency (e.g. means) or other basic estimates (e.g. regression coefficient) AND variation (e.g. standard deviation) or associated estimates of uncertainty (e.g. confidence intervals)
☐	☒	For null hypothesis testing, the test statistic (e.g. *F*, *t*, *r*) with confidence intervals, effect sizes, degrees of freedom and *P* value noted<br>*Give P values as exact values whenever suitable.*
☒	☐	For Bayesian analysis, information on the choice of priors and Markov chain Monte Carlo settings
☒	☐	For hierarchical and complex designs, identification of the appropriate level for tests and full reporting of outcomes
☒	☐	Estimates of effect sizes (e.g. Cohen's *d*, Pearson's *r*), indicating how they were calculated

Our web collection on statistics for biologists contains articles on many of the points above.

Software and code

Policy information about availability of computer code

Data collection	All described in the methods section of paper.
Data analysis	Graphpad Prism 8

For manuscripts utilizing custom algorithms or software that are central to the research but not yet described in published literature, software must be made available to editors and reviewers. We strongly encourage code deposition in a community repository (e.g. GitHub). See the Nature Portfolio guidelines for submitting code & software for further information.

Data

Policy information about availability of data

All manuscripts must include a data availability statement. This statement should provide the following information, where applicable:

- Accession codes, unique identifiers, or web links for publicly available datasets
- A description of any restrictions on data availability
- For clinical datasets or third party data, please ensure that the statement adheres to our policy

The raw data have been deposited in Figshare (https://figshare.com/articles/figure/Comparison_of_10_emerging_SARS-CoV-2_Variants_infectivity_animal_tropism_and_antibody_neutralization/14526894).

Field-specific reporting

Please select the one below that is the best fit for your research. If you are not sure, read the appropriate sections before making your selection.

- [x] Life sciences
- [ ] Behavioural & social sciences
- [ ] Ecological, evolutionary & environmental sciences

For a reference copy of the document with all sections, see nature.com/documents/nr-reporting-summary-flat.pdf

Life sciences study design

All studies must disclose on these points even when the disclosure is negative.

Sample size	Not applicable.
Data exclusions	No data was excluded.
Replication	Not applicable.
Randomization	Not applicable.
Blinding	Not applicable.

Reporting for specific materials, systems and methods

We require information from authors about some types of materials, experimental systems and methods used in many studies. Here, indicate whether each material, system or method listed is relevant to your study. If you are not sure if a list item applies to your research, read the appropriate section before selecting a response.

Materials & experimental systems

n/a	Involved in the study	
[ ]	[x]	Antibodies
[ ]	[x]	Eukaryotic cell lines
[x]	[ ]	Palaeontology and archaeology
[ ]	[x]	Animals and other organisms
[ ]	[x]	Human research participants
[x]	[ ]	Clinical data
[x]	[ ]	Dual use research of concern

Methods

n/a	Involved in the study	
[x]	[ ]	ChIP-seq
[ ]	[x]	Flow cytometry
[x]	[ ]	MRI-based neuroimaging

Antibodies

Antibodies used	mAbs A261-262, A247 and 76A were from Professor Linqi Zhang of Tsinghua University. mAb H00S022 was from Sino Biological Company. mAbs 1F9, 7B8, 4E5, 2H10, 10D12, 10F9 and 9G11 were from Beijing Biocytogen Co. mAb X593 was from Prof. sunney Xie of Peking University. mAb CB6 was provided by Prof. Jinghua Yan from the Institute of Microbiology, Chinese Academy of Sciences.MW06 anti-spike antibody was from Kohnoor Science & Technology Co., Beijing, China. FITC-labeled goat anti-human IgG (ZF-0308) was from Zhongshan Jinqiao, Beijing, China.
Validation	The antibodies were authenticated by provides.

Eukaryotic cell lines

Policy information about cell lines

Cell line source(s)	Three human cell lines Huh-7, Calu-3, and 293T was from Japanese Collection of Research Bioresources (Cat: 0403) and American Type Culture Collection (ATCC, Cat: HTB-55 and CRL-3216). Two monkey cell lines LLC-MK2 and Vero were from ATCC (Cat: CCL-7 and CCL-81). 293T-hACE2, 293T-hACE2-Furin, 293T-hACE2-TMPRSS2 and 293T-hACE2-Cathepsin L overexpressing cells were human ACE2, Furin, TMPRSS2, and Cathepsin L-stably expressing 293T cells. Receptor-transiently overexpressing cells were prepared by transfecting 293T cells with plasmids containing ACE2 from different species.
Authentication	The authentication of cells have been confirmed using STR method
Mycoplasma contamination	All cell lines were tested negative for mycoplasma contanmination
Commonly misidentified lines (See ICLAC register)	None

Animals and other organisms

Policy information about studies involving animals; ARRIVE guidelines recommended for reporting animal research

Laboratory animals	The Animals were handled in accordance with the protocol and guidelines for laboratory animal care and use. Mice were immunized with purified Trimer protein with aluminum adjuvant (20μg per mouse, once per week for 3 weeks). Serum samples were collected at 4 weeks after the third immunization. Serum samples from 10 mice of each group were pooled to produce combined samples. Each two mice were 483 combined to make one sample. Guinea pigs were immunized with SARS-CoV-2-Spike plasmid at 200μg per guinea pig or peudotyped virus at 6×105 TCID50 per guinea pig(once every 2 weeks for 6 weeks). Serum samples from five guinea pigs in each group were collected 28 days after the third immunization. Horses were immunized with SARS-CoV-2 RBD protein plus Freund's incomplete adjuvant (once every 10 days for 30 days) at doses of 3mg, 6mg and 12mg. Serum samples were collected at 1 week after the third immunization.
Wild animals	None
Field-collected samples	None
Ethics oversight	The study protocol was approved by the Animal Care and Use Committee at the National Institutes for Food and Drug Control (NIFDC).

Note that full information on the approval of the study protocol must also be provided in the manuscript.

Human research participants

Policy information about studies involving human research participants

Population characteristics	Twenty convalescence serum samples were collected from patients with COVID-19, 2-3 months after SARS-CoV-2 infection. Of these 10 samples, 5 were from D614G reference strain-infected patients, four were from B.1.1.7-infected patients, and three were from B.1.351-infected patients.
Recruitment	The serum samples were collected from convalescent patients of COVID-19.
Ethics oversight	All patients provided written informed consent to participate in the study.

Note that full information on the approval of the study protocol must also be provided in the manuscript.

Flow Cytometry

Plots

Confirm that:

- [x] The axis labels state the marker and fluorochrome used (e.g. CD4-FITC).
- [x] The axis scales are clearly visible. Include numbers along axes only for bottom left plot of group (a 'group' is an analysis of identical markers).
- [x] All plots are contour plots with outliers or pseudocolor plots.
- [x] A numerical value for number of cells or percentage (with statistics) is provided.

Methodology

Sample preparation	293T cells were transfected using the same procedure as for packaging the pseudotyped virus. The medium was removed after transfection for 36 hours, following which the cells were digestion to produce a single-cell suspension, washed once with PBS, and resuspended with PBS solution containing 1% BSA at 1×106 cells/tube.
Instrument	FACS CantoTM II
Software	FlowJo-V10
Cell population abundance	10,000 cells were collected for each group. Over 9,000 were in the post-gated fractions.
Gating strategy	Cells were preliminary gated by FSC/SSC only.

- [x] Tick this box to confirm that a figure exemplifying the gating strategy is provided in the Supplementary Information.
